# Greenspace and Land Cover Diversity During Pregnancy in a Rural Region, and Associations With Birth Outcomes

**DOI:** 10.1029/2023GH000905

**Published:** 2024-01-23

**Authors:** Jonathan W. Chipman, Xun Shi, Diane Gilbert‐Diamond, Camilo Khatchikian, Emily R. Baker, Mark Nieuwenhuijsen, Margaret R. Karagas

**Affiliations:** ^1^ Department of Geography Dartmouth College Hanover NH USA; ^2^ Department of Epidemiology Geisel School of Medicine at Dartmouth Lebanon NH USA; ^3^ Children's Environmental Health and Disease Prevention Research Center at Dartmouth Hanover NH USA; ^4^ Department of Obstetrics and Gynecology Dartmouth Hitchcock Medical Center Lebanon NH USA; ^5^ Centre for Research in Environmental Epidemiology ISGlobal Barcelona Spain

## Abstract

Beneficial effects on health outcomes have been observed from exposure to spaces with substantial green vegetation (“greenspace”). This includes studies of greenspace exposure on birth outcomes; however, these have been conducted largely in urban regions. We characterized residential exposure to greenspace and land cover diversity during pregnancy in rural northern New England, USA, investigating whether variation in greenspace or diversity related to newborn outcomes. Five landscape variables (greenspace land cover, land cover diversity, impervious surface area, tree canopy cover, and the Normalized Difference Vegetation Index) were aggregated within six circular zones of radii from 100 to 3,000 m around residential addresses, and distance to conservation land was measured, providing a total of 31 greenspace and diversity metrics. Four birth outcomes along with potentially confounding variables were obtained from 1,440 participants in the New Hampshire Birth Cohort Study. Higher greenspace land cover up to 3,000 m was associated with larger newborn head circumference, while impervious surface area (non‐greenspace) had the opposite association. Further, birth length was positively associated with land cover diversity. These findings support beneficial health impacts of greenspace exposure observed in urban regions for certain health outcomes, such as newborn head circumference and length but not others such as birthweight and gestational age. Further our results indicate that larger radius buffer zones may be needed to characterize the rural landscape. Vegetation indices may not be interchangeable with other greenspace metrics such as land cover and impervious surface area in rural landscapes.

## Key Points

- Greenspace land cover and diversity associated with larger head circumference and longer birth length, respectively
- Our findings were seen for larger buffer zones and different outcomes than reported in urban studies
- Results for measures of biodiversity suggest further research using these measures

## 1 Introduction

Studies over the past decade have reported that exposure to landscape features, particularly greenspace, can have a positive impact on human health (Amoly et al., 2014; Barton & Pretty, 2010; Dadvand & Nieuwenhuijsen, 2019; de Keijzer et al., 2020; Houlden et al., 2018; Maas et al., 2006; Markevych et al., 2017; Twohig‐Bennett & Jones, 2018). Properties of greenspace such as the presence of “natural” features and more specifically green vegetation are hypothesized to provide benefits to human health for individuals who passively or actively interact with the landscape. While the reasons for this are not fully understood, they range from physical health (Dadvand et al., 2012; Jiang et al., 2020; Klompmaker et al., 2018; Maas et al., 2006; Wolch et al., 2011) to mental and emotional health (Barton & Pretty, 2010; Helbich et al., 2018; Holt et al., 2019; Houlden et al., 2018; Korpela et al., 2014), and cognitive development (Amoly et al., 2014; Dadvand et al., 2015). The impacts of greenspace on health have been observed across all ages, from birth (Dadvand et al., 2012) and childhood to old age (de Keijzer et al., 2020).

In evaluating the quality of many greenspace studies (*n* = 125), the majority did not explicitly define greenspace and how it was derived (Taylor & Hochuli, 2017). Differing definitions or sources of data on greenspace can influence study results (Klompmaker et al., 2018). Some studies use self‐reported time spent in green or natural environments, leaving the definition open to interpretation (Bloemsma et al., 2018; Holt et al., 2019; Korpela et al., 2014). Others use quantitative landscape metrics to represent greenspace, such as proximity to parks or other outdoor recreational areas (Amoly et al., 2014; Wolch et al., 2011), and various formal land cover/land use classifications (Helbich et al., 2018; Klompmaker et al., 2018; Maas et al., 2006), or numerical vegetation indices derived from multispectral remote sensing images (Amoly et al., 2014; Dadvand et al., 2012, 2015; Jiang et al., 2020).

Research primarily has examined densely populated urban areas where greenspace is scarce. Dadvand and Nieuwenhuijsen (2019) summarized the nature of urban greenspace and the mechanisms by which greenspace may affect health. These included reduced stress from air pollution, noise and heat, as well as social cohesion and increased positive social interactions, physical activity, and microbiodiversity/environmental microbial input (Dadvand & Nieuwenhuijsen, 2019).

A growing number of studies have focused on the effects of greenspace exposure on pregnancy and birth outcomes. A review of the literature (Lee et al., 2020) identified 89 studies, of which 10 qualified for inclusion in a meta‐analysis of the association between birth outcomes and a satellite derived vegetation index. Surrounding greenness was found to be associated with better pregnancy outcomes in the meta‐analysis; of the 10 included studies, seven were in urban areas, two in mixed urban/suburban/rural areas, and one was a review of multiple prior studies (Lee et al., 2020). The preponderance of greenspace research on urban areas raises the questions: Does the health impact of greenspace continue to increase linearly across the full range of potential values of greenspace? Or does it saturate at higher levels of greenspace, yielding decreasing benefits beyond some threshold? In a study across the urban‐to‐rural gradient in Pennsylvania, USA, positive effects of greenspace on birth outcomes were found in urban areas, but not within more rural townships (Casey et al., 2016). Evidence of persistent positive effects within a rural region was reported for the Henan Rural Cohort in Henan, China (Jiang et al., 2020). In Queensland, Australia, Vilcins et al. (2021) reported that maternal residential proximity to green cover was associated with higher birthweight, and dry cover or bare earth with lower birthweight, in both urban and rural areas (Vilcins et al., 2021).

**Figure 1:**
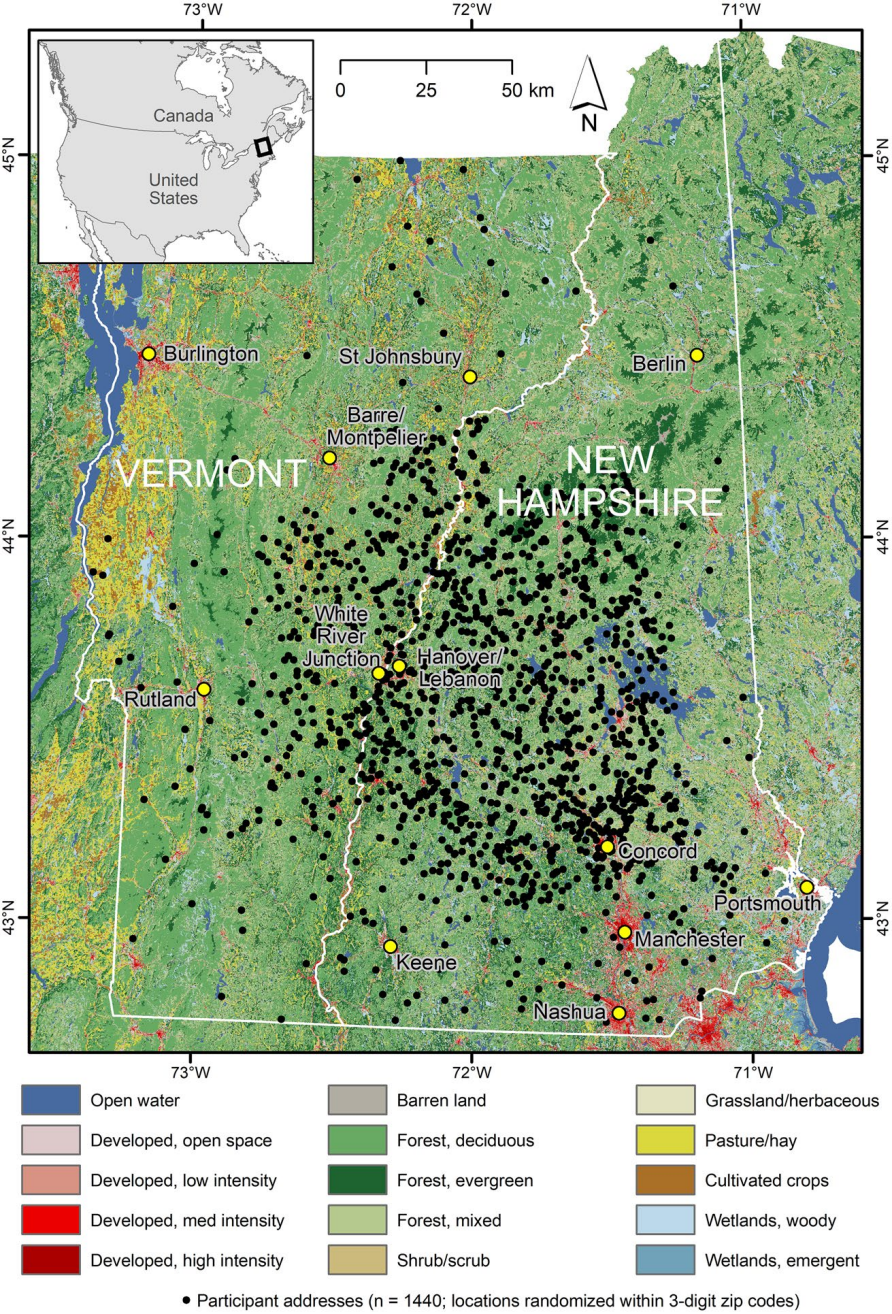
Map of the study region, showing NLCD land cover classes and participant residential locations (masked by randomization within 3‐digit zip code tabulation areas (ZCTAs); low‐population ZCTAs are merged with neighboring ZCTAs). Selected regionally significant cities and towns are indicated by yellow circles.

In light of these questions, we sought to further address the role of greenspace on birth outcomes (birth weight, length, head circumference and gestational age) among residents of two predominantly rural states—New Hampshire and Vermont—ranking 11th and 2nd most rural in the US respectively (US Census Bureau, 2012). As shown in Figure 1, land cover in the region is a mosaic of forest types interspersed with small urban areas and relict agricultural lands primarily located in river valleys. It thus differs widely from both the urban landscapes typical of most greenspace research and from the densely farmed landscape of the Henan Rural Cohort (Jiang et al., 2020).

We considered six different conceptualizations of greenspace—based on land cover, impervious surfaces, tree cover, vegetation indices, diversity of land cover, and proximity to conservation/protected lands—as well as a range of spatial scales extending across those employed in prior studies of greenspace. We hypothesized that this comprehensive approach, covering a range of both spatial scales and definitional representations of greenspace, would help to identify whether a continued impact on birth outcomes exists with variations of greenspace in the context of high overall greenspace exposure.

## 2 Materials and Methods

Our study group comprised 1,440 participants with geocodable addresses recruited during the 2009–2018 period from the New Hampshire Birth Cohort Study (NHBCS) (Gilbert‐Diamond et al., 2011, 2016). The NHBCS was designed to study the effects of environmental exposures on maternal and child health, including metals and metalloids (Romano et al., 2019; Shi et al., 2015; Signes‐Pastor et al., 2019), diet (Emond et al., 2018; Gilbert‐Diamond et al., 2011), air quality (Fleisch, Rokoff, et al., 2020; Fleisch, Seshasayee, et al., 2020), and other pathways. Prospective participants meeting the cohort criteria (ages 18–45, singleton pregnancy, literate in English, in homes with private water wells, and not planning to move) were recruited from prenatal clinics in New Hampshire (Emond et al., 2018; Gilbert‐Diamond et al., 2016). Maternal sociodemographic information (e.g., age at enrollment, race/ethnicity, highest level of education attainment, type of insurance), lifestyle factors (e.g., amount of physical activity and cigarette smoking), pregnancy history and health information was collected via questionnaires, prenatal and delivery records (e.g., pre‐pregnancy BMI, delivery mode). Birth anthropometric data were abstracted from infants' delivery records. Gestational age was determined from ultrasounds or if unavailable based on the last menstrual period record in the prenatal records. Additionally, maternal urine samples were obtained at approximately 24–28 weeks gestation and analyzed for arsenic species as previously described (Gilbert‐Diamond et al., 2011).

Greenspace‐related landscape metrics for this study included the following:

1. *Land cover classes representative of undeveloped open space (LC).* These data were obtained from the US National Land Cover Database 2011 (NLCD) at 1 arcsecond grid spacing, or approximately 30 m (Homer et al., 2015; Wickham et al., 2017). All classes except 22–24 (low/medium/high intensity developed) were coded as greenspace. The regional distribution of NLCD land cover is shown in Figure 1.
2. *Impervious surface area (IS)*, inversely related to greenspace. These data were also obtained from the NLCD archive, at the same 1‐arcsecond grid spacing. Each grid cell contains the fraction of impervious surfaces, from 0 to 1 (Wickham et al., 2020).
3. *Tree canopy cover (TC).* As with (1) and (2) above, these were obtained from the NLCD archive at 1 arcsecond spacing. Cell values are the fraction of tree canopy cover (Coulston et al., 2012; Ruefenacht et al., 2015).
4. *The Landsat‐8 Normalized Difference Vegetation Index (NDVI).* The ensemble of all cloud‐free pixels from 382 Landsat‐8 multispectral images during the seasonal window of June 1 to 30 September 2013–2016, was used to compute summer mean NDVI (Lillesand et al., 2015; Rouse et al., 1974; Tucker, 1979). The images have a nominal spatial resolution of 30 m. Pixel‐level filtering based on the quality assurance flags was used to remove pixels categorized as clouds or cloud shadows. Areas classified as water or emergent wetland in the NLCD data were excluded from the NDVI calculations, due to the low values of water in the NDVI metric.
5. *Distance to conservation land (Dist_CL)*, inversely related to greenspace. Vector geospatial databases of conservation lands were obtained from the states of New Hampshire (NH GRANIT, 2020) and Vermont (Vermont Center for Geographic Information (VCGI), 2020). The distance in km to the nearest parcel of designated conservation land was calculated for each address, as described below.
6. *Diversity of land cover (Div).* The Shannon Diversity index was calculated from the proportions of all NLCD land cover classes within each of the six buffer distances. While not directly representative of greenspace, land cover diversity is used here as a proxy for environmental biodiversity, which has been suggested to impact human health via the microbiome (Hanski et al., 2012; Roslund et al., 2020).

To extract data from these greenspace sources, participant addresses from the NHBCS data set (all located in the US states of New Hampshire and Vermont; Figure 1) were geocoded. Circular buffer zones with radii of 100, 250, 500, 1,000, 2,000, and 3,000 m were constructed around all 1,440 latitude/longitude points and used to extract greenspace land cover fraction (“LC”), impervious surface area fraction (“IS”), tree canopy cover fraction (“TC”), and mean NDVI vegetation index (“VI”) within each buffer. These buffer distances were chosen to match prior work elsewhere, which has varied across scales from under 100 m to over 3,000 m (Amoly et al., 2014; Maas et al., 2006; Zhan et al., 2020; Zhao et al., 2022). In recent years there has been a shift toward inclusion of larger buffer distances in studies of greenspace; two recent reviews show that 50% of papers in 2019–2022 included buffers of 1,000+ m, versus only 20% in 2012–2015 (Zhan et al., 2020; Zhao et al., 2022). For this study, the three smaller radii were included for consistency with the majority of prior studies, and the three larger to reflect the trend in the literature and to test the hypothesis that the spatial scale of the greenspace/health effect is coarser in rural areas. Distance to conservation land (“Dist_CL”) was calculated as the straight‐line distance (in km) from each participant's address to the nearest parcel of conservation land. Land cover diversity (“Div”) was calculated from the NLCD land cover data within each buffer distance, using the Shannon index (Equation 1):

(1) $$s=-\sum_{i=1}^{N}\left(p_{i}\;\ln\;p_{i}\right)$$

where *N* is the number of land cover classes present at a given site, *p*_*i*_ is the proportion of class *i* at that site, and *s* is the Shannon Diversity index value for the site. A site with uniform land cover has *s* = 0, while a site with heterogeneous land cover has *s* > 0, increasing with the number of classes.

**Figure 2:**
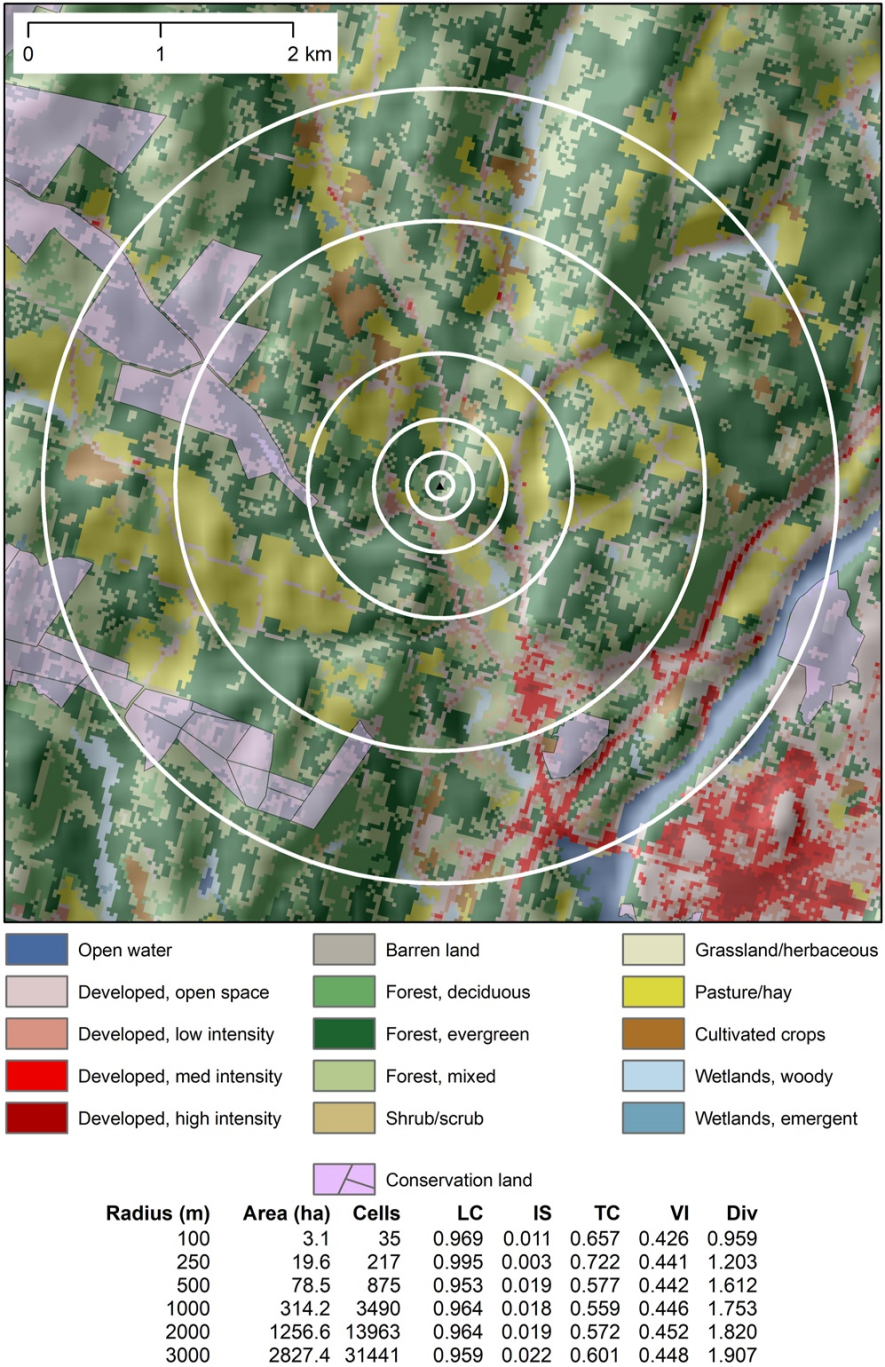
Six nested circular buffers around a sample location, with radii of 100, 250, 500, 1,000, 2,000, and 3,000 m. Basemap shows NLCD land cover (Homer et al., 2015) with shaded relief. Greenspace metrics for each buffer are listed in the table. Sample location is not an actual address from the study.

An example of the concentric buffer zones, with land cover and conservation land data, is given in Figure 2. Buffer generation, extraction of data within the buffers, and calculation of distances were done in ArcGIS Desktop (ESRI, 2016) and QGIS; calculation of NDVI from the Level‐2 Landsat spectral data was done in the Google Earth Engine (Gorelick et al., 2017). The result was a collection of 31 greenspace metrics (five buffer‐extracted variables *X* six buffer radii, plus one distance measurement) for each NHBCS address.

Correlations among all pairs of greenspace metrics were calculated, to examine the consistency of greenspace‐related indicators both as a function of scale (buffer radius) and data source (LC, IS, TC, VI, Dist_CL, Div). Correlations among the non‐greenspace independent variables (health metrics), and between the greenspace and health metrics, were also examined.

Multivariate regression was used to model the relationship between each greenspace metric and each of the four dependent variables (length, weight, and head circumference *z*‐scores following World Health Organization standards, and gestational age in weeks) controlling for the potential confounding effects of maternal age, parity, history of prior pregnancy, maternal educational level (high school or less, junior college graduate or some college, college graduate, any post‐graduate schooling), maternal insurance (private or public/other), self‐reported exercise during pregnancy (yes/no), birth season, smoking history (yes/no), child's sex, maternal BMI, maternal race/ethnicity, and maternal prenatal total urinary arsenic. Delivery type (vaginal or c‐section) was also included as a potentially confounding variable in models of head circumference. Robust regression (Hampel et al., 2011; Huber, 2004; Marazzi, 1993) as implemented in the Robust Fitting of Linear Models (rlm) algorithm in the R statistical computing environment was used to model the dependent variables, because of its robust handling of outliers. In this algorithm, the model is fit by iterated re‐weighted least squares (IWLS). Each model was embedded in a 10‐fold cross validation process (using the Caret package in R), to provide a better estimate of model performance on validation data. Missing covariates were imputed using the Multivariate Imputation by Chained Equations algorithm (MICE) in R. This imputation method estimates missing values in a multivariate data set via predictive mean matching for continuous data, and logistic regression, polytomous logistic regression, and proportional odds for binary and categorical data (Van Buuren & Groothuis‐Oudshoorn, 2011). Ten replicates of the imputation process were conducted, and the outputs were aggregated by taking the median of the 10 values for each data point of a continuous variable and the mode of the 10 for a binary or categorical variable. To account for the false discovery rate (FDR), the nominal *a* = 0.05 threshold of significance was adjusted for each model using the Benjamini‐Hochberg approach (Benjamini & Hochberg, 1995; Benjamini & Yekutieli, 2001). Finally, a sensitivity analysis was performed using alternative methods, deleting all birth cohort records with any missing values for any covariate (rather than imputation), and using the generalized linear models (glm) algorithm in R, in place of robust regression (rlm). More details are provided in Supporting Information S1 (Sensitivity Analysis: Alternative Methods and Data). Supporting Information S1 also includes the results of models for two alternative choices for birth outcomes: preterm births and low birth weights, both using binomial versions of the glm method (Table S2 in Supporting Information S1).

## 3 Results

Overall 96% of study participants reported their race as non‐Hispanic white (Table 1). This is consistent with the regional demographics and selection of those with a private water system. Also, about one third (31%) reported that they did not receive a college degree (Table 1). Approximately 53% of participants self‐reported engaging in exercise, 12% had ever smoked, and 6% reported having smoked during pregnancy (Table 1). The vast majority (84%) had private insurance (Table 1). For the birth outcomes, median *z*‐scores for height, weight, and head circumference were all slightly positive (Table 1).

**Table 1:** Summary Statistics for Selected Characteristics and Birth Outcomes

Variable			Imputed
Length *z*‐score at birth	0.65	{−0.40–1.64}	0.0%
Weight *z*‐score at birth	0.30	{−0.35–0.91}	0.0%
Head circumference *z*‐score at birth	0.42	{−0.36–1.21}	0.0%
Gestational age (weeks)	39.57	{38.72–40.54}	0.0%
Maternal age	31.43	{28.10–34.73}	0.0%
Maternal BMI (log)	1.39	{1.34–1.46}	2.6%
Urinary arsenic (log)	0.61	{0.35–0.79}	25.3%
Delivery type			0.2%
Vaginal	996	[69.2]	
C‐section	444	[30.8]	
Parity			0.6%
0	617	[42.8]	
1	520	[36.1]	
2	200	[13.9]	
3+	103	[7.2]	
Ever pregnant			0.9%
No	420	[29.2]	
Yes	1,020	[70.8]	
Maternal education level			12.8%
High school or less	167	[11.6]	
Junior college graduate or some college or technical school	284	[19.7]	
College graduate	577	[40.1]	
Postgraduate schooling	412	[28.6]	
Maternal insurance			14.2%
Private	1,207	[83.8]	
Public or other	233	[16.2]	
Maternal exercise during pregnancy			22.8%
No	682	[47.4]	
Yes	758	[52.6]	
Birth season			0.0%
Spring	391	[27.2]	
Summer	397	[27.6]	
Fall	341	[23.7]	
Winter	311	[21.6]	
Smoking status			14.2%
Never	1,263	[87.7]	
Past or current	177	[12.3]	
Baby male			0.0%
No	713	[49.5]	
Yes	727	[50.5]	
Maternal race/ethnicity			0.0%
White and not Hispanic/Latino	1,376	[95.6]	
All other	64	[4.4]	

*Note.* Data are presented as median {interquartile range} or number [%].

As expected, our cohort was predominantly rural, with greenspace land cover fractions typically above 90%, impervious surface fractions mostly below 5%, tree canopy fraction mostly in the 40%–70% range, and mean summer NDVI values between 0.18 and 0.48. The region is well endowed with protected/conservation lands, with the median distance from NHBCS addresses to the nearest parcel of such lands approximately 0.5 km (Table 2).

**Table 2:** Summary Statistics for Greenspace Metrics

Metric	Radius (m)	Code	Min	25%	Mean	Median	75%	Max
Greenspace land cover fraction (0–1)	100	LC_0100	0.000	0.829	0.865	0.943	1.000	1.000
	250	LC_0250	0.005	0.899	0.917	0.963	0.995	1.000
	500	LC_0500	0.009	0.923	0.939	0.972	0.991	1.000
	1,000	LC_1000	0.028	0.938	0.950	0.973	0.990	1.000
	2,000	LC_2000	0.165	0.948	0.957	0.975	0.989	1.000
	3,000	LC_3000	0.223	0.950	0.959	0.974	0.987	1.000
Impervious surface fraction (0–1)	100	IS_0100	0.000	0.007	0.067	0.034	0.080	0.689
	250	IS_0250	0.000	0.006	0.042	0.019	0.046	0.699
	500	IS_0500	0.000	0.006	0.031	0.014	0.037	0.711
	1,000	IS_1000	0.000	0.006	0.026	0.013	0.030	0.683
	2,000	IS_2000	0.000	0.006	0.022	0.012	0.026	0.565
	3,000	IS_3000	0.000	0.007	0.021	0.013	0.026	0.518
Tree canopy cover fraction (0–1)	100	TC_0100	0.081	0.362	0.424	0.410	0.477	0.872
	250	TC_0250	0.114	0.487	0.578	0.600	0.689	0.881
	500	TC_0500	0.150	0.551	0.622	0.639	0.710	0.877
	1,000	TC_1000	0.175	0.589	0.646	0.663	0.720	0.866
	2,000	TC_2000	0.222	0.618	0.663	0.675	0.719	0.834
	3,000	TC_3000	0.252	0.627	0.670	0.678	0.721	0.825
Normalized difference vegetation index (NDVI) (−1 to 1)	100	VI_0100	0.175	0.362	0.382	0.386	0.405	0.475
	250	VI_0250	0.170	0.377	0.394	0.397	0.413	0.470
	500	VI_0500	0.200	0.386	0.400	0.404	0.417	0.457
	1,000	VI_1000	0.213	0.392	0.404	0.406	0.419	0.474
	2,000	VI_2000	0.235	0.396	0.407	0.408	0.420	0.471
	3,000	VI_3000	0.256	0.399	0.409	0.409	0.420	0.461
Distance to conservation lands (km)		Dist_CL	0.000	0.222	0.699	0.520	0.983	4.231
Land cover diversity (Shannon diversity index)	100	Div_0100	0.000	0.923	1.150	1.185	1.404	2.003
	250	Div_0250	0.228	1.236	1.448	1.461	1.690	2.198
	500	Div_0500	0.550	1.426	1.611	1.628	1.814	2.257
	1,000	Div_1000	0.785	1.554	1.715	1.708	1.892	2.434
	2,000	Div_2000	0.898	1.636	1.787	1.779	1.940	2.414
	3,000	Div_3000	1.058	1.679	1.816	1.816	1.948	2.446

*Note.* LC, IS, and TC are unitless fractions in the range 0–1; NDVI (also unitless) has a range of −1 to +1; Dist_CL has units of km (0+); Div (unitless) has values 0+.

Correlations among the greenspace metrics were highest between adjacent scales of buffer radii (e.g., 100 and 250 m, or 500 and 1,000 m) within the same data source (LC, IS, TC, or VI). There was also a strong inverse correlation between the greenspace (non‐developed) land cover fraction and impervious surface fraction; however, there are no other pairs of data sources (other than LC‐IS) for which any combination of buffers between the two sources has correlations with absolute values >0.80 (Figure S1 in Supporting Information S1).

Each model was subjected to 10‐fold cross validation. The *β*^ estimates for the greenspace metric in each model are listed in Table 3; models with greenspace p‐values below 0.05 and 0.01 are highlighted. When interpreting the sign of *β*^ values, note that the metrics IS (impervious surface fraction) and Dist_CL functionally are considered the converse of greenspace.

**Table 3:** Values of *β*^ and Its 95% Confidence Interval for Each Combination of Health Outcome and Greenspace Metric

	Length *z*‐score	Weight *z*‐score	Head circumference *z*‐score	Gestational age
LC_0100	−0.18 (−0.56, 0.2)	−0.01 (−0.28, 0.26)	−0.05 (−0.4, 0.29)	−0.15 (−0.53, 0.22)
LC_0250	−0.07 (−0.61, 0.47)	0.07 (−0.32, 0.45)	0.1 (−0.41, 0.6)	0.06 (−0.49, 0.61)
LC_0500	0.12 (−0.57, 0.8)	0.15 (−0.34, 0.63)	0.58 (−0.09, 1.25)	0.4 (−0.33, 1.14)
LC_1000	0.25 (−0.55, 1.05)	0.21 (−0.36, 0.77)	0.81 (−0.02, 1.64)	0.77 (−0.14, 1.68)
LC_2000	0.13 (−0.81, 1.07)	0.01 (−0.66, 0.67)	1.08 (0.01, 2.15)	0.96 (−0.21, 2.14)
LC_3000	−0.12 (−1.13, 0.9)	−0.08 (−0.8, 0.64)	1.32 (0.09, 2.56)	1 (−0.35, 2.35)
IS_0100	0.38 (−0.39, 1.14)	−0.18 (−0.73, 0.36)	−0.04 (−0.72, 0.65)	0.04 (−0.7, 0.78)
IS_0250	0.21 (−0.85, 1.27)	−0.31 (−1.06, 0.43)	−0.39 (−1.32, 0.54)	−0.31 (−1.33, 0.7)
IS_0500	−0.05 (−1.41, 1.3)	−0.47 (−1.43, 0.49)	−1.16 (−2.35, 0.03)	−0.83 (−2.14, 0.48)
IS_1000	−0.15 (−1.81, 1.5)	−0.61 (−1.78, 0.56)	−1.42 (−2.87, 0.03)	−1.24 (−2.84, 0.36)
IS_2000	0.39 (−1.72, 2.5)	−0.26 (−1.76, 1.23)	−1.8 (−3.66, 0.06)	−1.48 (−3.51, 0.55)
IS_3000	1.02 (−1.41, 3.44)	−0.11 (−1.83, 1.62)	−2.24 (−4.38, −0.1)	−1.46 (−3.81, 0.88)
TC_0100	0.16 (−0.47, 0.78)	0.2 (−0.25, 0.64)	0.58 (0.01, 1.14)	0.39 (−0.23, 1)
TC_0250	−0.26 (−0.74, 0.23)	−0.16 (−0.51, 0.18)	−0.5 (−0.93, −0.07)	−0.45 (−0.93, 0.03)
TC_0500	−0.54 (−1.14, 0.06)	−0.26 (−0.68, 0.17)	−0.26 (−0.81, 0.29)	−0.34 (−0.94, 0.26)
TC_1000	−0.47 (−1.16, 0.23)	−0.26 (−0.75, 0.23)	0 (−0.65, 0.65)	−0.12 (−0.83, 0.59)
TC_2000	−0.46 (−1.29, 0.37)	−0.36 (−0.95, 0.22)	−0.07 (−0.88, 0.73)	0.26 (−0.62, 1.14)
TC_3000	−0.49 (−1.41, 0.43)	−0.35 (−1.01, 0.3)	0.05 (−0.86, 0.97)	0.41 (−0.59, 1.41)
VI_0100	−1.28 (−3.05, 0.49)	−0.78 (−2.03, 0.47)	−1.18 (−2.99, 0.62)	−0.97 (−2.93, 0.99)
VI_0250	−1.35 (−3.33, 0.62)	−0.77 (−2.17, 0.63)	−1.09 (−3.22, 1.04)	−0.3 (−2.62, 2.03)
VI_0500	−1.21 (−3.37, 0.95)	−0.63 (−2.16, 0.9)	0.46 (−1.97, 2.9)	0.68 (−1.98, 3.34)
VI_1000	−0.54 (−2.85, 1.77)	−0.34 (−1.98, 1.29)	1.25 (−1.49, 3.99)	1.57 (−1.43, 4.57)
VI_2000	−1.02 (−3.52, 1.48)	−0.79 (−2.57, 0.99)	1.75 (−1.52, 5.02)	0.97 (−2.62, 4.56)
VI_3000	−1.05 (−3.7, 1.59)	−0.93 (−2.8, 0.95)	2.01 (−1.66, 5.67)	1.61 (−2.4, 5.63)
Dist_CL	−0.09 (−0.2, 0.03)	−0.03 (−0.11, 0.05)	0.01 (−0.09, 0.11)	0.04 (−0.07, 0.15)
Div_0100	0.09 (−0.12, 0.3)	0.02 (−0.13, 0.17)	−0.1 (−0.29, 0.09)	0.09 (−0.11, 0.3)
Div_0250	0.16 (−0.07, 0.38)	0.01 (−0.15, 0.17)	−0.03 (−0.23, 0.18)	0.15 (−0.07, 0.38)
Div_0500	0.25 (0, 0.5)	0.06 (−0.12, 0.24)	0.06 (−0.17, 0.29)	0.25 (−0.01, 0.5)
Div_1000	0.36 (0.07, 0.65)	0.05 (−0.16, 0.25)	−0.04 (−0.31, 0.23)	0.07 (−0.23, 0.36)
Div_2000	0.47 (0.15, 0.79)	0.07 (−0.15, 0.3)	−0.16 (−0.44, 0.13)	−0.06 (−0.39, 0.28)
Div_3000	0.5 (0.17, 0.84)	0.05 (−0.18, 0.29)	−0.16 (−0.46, 0.13)	−0.22 (−0.58, 0.14)

*Note.* Dark and light highlighting indicate *p*‐value <0.01 and <0.05 respectively, prior to adjustment for false discovery rate.

Overall, we did not observe consistent associations with gestational age (Table 3). Head circumference *z* scores increased with greenspace land cover (*β* = 1.32 *z* or 1.62 mm per 0.1 unit increase in land cover fraction; 95% CI 0.09, 2.56 *z* or 0.11, 3.15 mm; *p* = 0.036) and decreased with impervious surface area at 3,000 m radius (*β* = −2.24 *z* or −2.76 mm per 0.1 unit increase in impervious surface fraction; 95% CI ‐4.38, −0.10 *z* or −5.39, 0.12 mm; *p* = 0.04) (Table 3). Length *z* scores increased with greater land cover diversity (*β* = 0.5 *z* or 0.94 mm per 0.1 unit increase in diversity; 95% CI 0.17, 0.84 *z* or 0.32, 1.57 mm; *p* = 0.003). Details on these models are provided in Table S1 of Supporting Information S1. Due to the number of comparisons, none of our models exceeded the adjusted threshold for significance with the Benjamini‐Hochberg FDR correction.

## 4 Discussion

Using data from the rural birth cohort from northern New England, we assessed the relationship between maternal residential exposure to greenspace and birth outcomes. Greenspace was characterized using multiple metrics across a range of six spatial scales. Correlations among the greenspace metrics were generally low, supporting the need to use multiple measures (land cover, impervious surface area, tree canopy cover, NDVI, and distance to conservation land) as they may indicate different aspects of greenspace exposure. Amount of impervious surface area within a 3,000 m buffer zone was inversely related to newborn head circumference, while greenspace land cover within 2,000–3,000 m was positively related. Land cover diversity was positively related to birth length within the 1,000–3,000 m distances. No consistent associations were observed for gestational age or weight *z*‐score (Table 3), nor for binomial models of variables related to these two (preterm birth [yes/no] and low birth weight [yes/no]) (Table S2 in Supporting Information S1).

The data sources used in our models encompass different concepts of greenspace: one based on generalized land cover classes, another on levels of impervious surfaces, a third on tree canopy cover, a fourth on satellite observations of local greenness, a fifth on proximity to protected or conservation lands, and a sixth on land cover diversity. They also cover a range of scales, from 100 to 3,000 m. While we found associations with certain anthropometric *z* scores, the explanatory value of the models (as indicated by *r* ^2^) was generally low. This may be in part because in a rural environment where greenspace is commonplace, variations in the amount of greenspace surrounding a pregnant person's residence are less influential on birth outcomes than in more frequently studied urban settings. Moreover, of the models highlighted in Table 3 based on *p* values, in a majority of cases the greenspace buffer radius was 1,000–3,000 m, larger than the distances used in most urban greenspace studies and suggestive that the spatial scale of landscape influences on health may be coarser in rural than urban areas. It is also possible that a lack of statistical power may have limited this study's ability to detect associations between greenspace and birth outcomes, given the sample size (1,440 participants) and variable but generally modest effect sizes reported by prior studies (Dzhambov et al., 2014).

Land cover diversity is used here as a coarse proxy for environmental biodiversity in the vicinity of an individual's residence. Prior work by Hanski et al. (2012) has shown the importance of environmental biodiversity for health, in particular via its impact on the microbiome (Hanski et al., 2012). Roslund et al. (2020) used biodiversity interventions around daycare centers to show increases in microbiome health and enhanced immune regulation among children (Roslund et al., 2020). Further research is needed to assess whether environmental biodiversity in rural areas has similar effects on commensal microbiota and human health.

**Figure 3:**
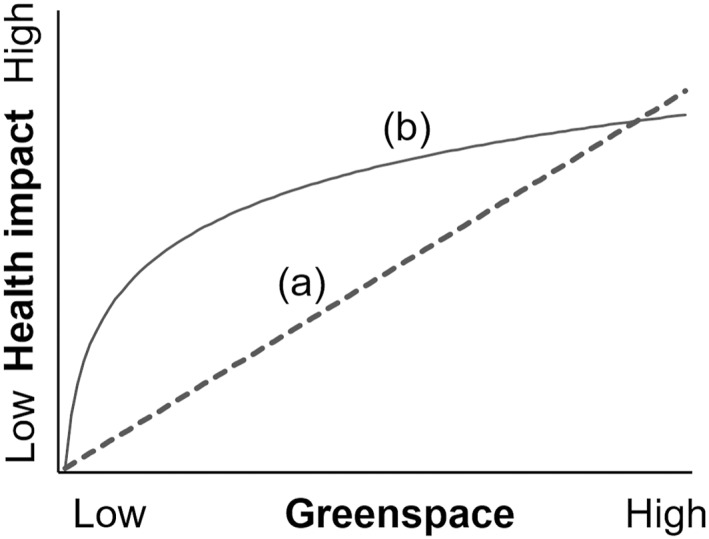
Two conceptual relationships between greenspace and health impact. (a) Health impact increases linearly over the entire range of greenspace fraction. (b) Health impact is highly responsive at low levels of greenspace but saturates when greenspace becomes widespread.

Our findings generally agree with those from the most similar prior work on greenspace and birth outcomes in a partially rural landscape in the northeastern USA (Casey et al., 2016). In a study covering an urban‐to‐rural gradient in Pennsylvania, associations between NDVI and preterm and SGA births were found in urban areas, but not within rural townships. Our study encompassed additional greenspace metrics and spatial scales and focused solely on variation within a rural region. Prior work over many years has clearly established that exposure to greenspace in highly developed urban landscapes (at the left end of the *X* axis in Figure 3) can have positive effects on health (Amoly et al., 2014; Barton & Pretty, 2010; Dadvand & Nieuwenhuijsen, 2019; de Keijzer et al., 2020; Houlden et al., 2018; Maas et al., 2006; Markevych et al., 2017; Twohig‐Bennett & Jones, 2018). Our study, along with that of Casey et al. (2016), situated at the right end of the *X* axis, suggests that rather than continuing to increase gradually as a function of greenspace exposure, the positive health impacts may reach a plateau where there is little further effect. In this context, virtually all the NHBCS participants may be receiving some benefits from the regionally widespread greenspace, relative to individuals in a densely built‐up urban core, but the marginal effect from further increases in proximal greenspace may not be evident. In contrast, where greenspace is already abundant, land cover diversity may become more significant than incremental increases in greenness. A similar pattern of decreasing benefits at high background levels of greenspace was found a meta‐analysis of 10 studies of mental health among adults from the UK (Barton & Pretty, 2010). In that analysis, even short periods of activity in greenspace were associated with positive mental health, but as time spent in greenspace increased, the incremental additional benefit approached zero, with no further marginal improvement in mental health outcomes. Further data integrating urban and rural environmental would be useful to evaluate this hypothesis.

Many previous studies of greenspace have used NDVI as a metric (Amoly et al., 2014; Dadvand et al., 2012, 2015; Jiang et al., 2020; Lee et al., 2020). Its popularity likely derives from the fact that it provides a robust, accurate, and well‐validated method to differentiate healthy green vegetation from unvegetated surfaces over large areas in satellite imagery. In an urban environment, NDVI thus provides a quick and easy way to separate greenspace from non‐greenspace. In a rural landscape like northern New England, NDVI may not be as suitable for use in differentiating among landscape units, because the differences among “green” areas are not necessarily mapped well onto the ways humans would categorize greenspace. For example, high NDVI values in our study may occur on well‐managed hayfields and high‐productivity northern hardwoods forest patches located on southeast‐facing slopes, but other forest stands have lower NDVI values. The idea that “greenspace = nature” breaks down when heavily managed landscapes can have higher values on a greenspace metric than some natural, unmanaged landscapes. In addition, dense forest cover in a region such as northern New England could conceivably have both positive and negative health effects; an example of the latter would be if the deep shade reduces maternal sun exposure and in turn vitamin D production. This could potentially adversely affect bone growth and hence birth length (Francis et al., 2018; Pérez‐López et al., 2015). However, this possibility requires further exploration in other populations living in similar environments. Moroever, in regions with cold winter climates, the physical and mental health effects of exposure to “whitespace” (snow‐covered greenspace) have yet to be investigated.

Other areas that have been identified as requiring further research include longitudinal studies of long‐term greenspace exposure and health (de Keijzer et al., 2020) and the consequences of unequal social access to greenspace and the urban/rural divide (Wolff et al., 2020). There is also a need for better understanding of how greenspace health pathways interact with other environmental factors in rural areas, in ways that may be quite different from those interactions in urban areas. For example, improved air quality is often cited as a benefit of greenspace in urban studies, while in rural regions (and greenspace‐surrounded) such as ours in a northern latitude, homes may be heated with woodstoves, with adverse health effects (Fleisch, Rokoff, et al., 2020; Fleisch, Seshasayee, et al., 2020).

In our analyses we did not consider behavior and active engagement in the landscape (time spent outdoors, amount of exercise) beyond the passive residential proximity to greenspace discussed here. Prior work has found that actual time spent in greenspace is not strongly linked to the objectively measured quantity of residential greenspace (Bloemsma et al., 2018). While nearly everyone in our study region has theoretical access to greenspace, the actual amount of time spent outdoors, and the personal, social, and environmental context and character of those outdoors activities vary. Thus, future work would benefit from considering these factors.

## 5 Conclusions

Prior work, predominantly from more urbanized areas or mixed areas, has identified associations between higher greenspace exposure with beneficial birth outcomes such as birth weight. Our findings suggest that variations within the relatively high levels of proximal greenspace in our rural study region may be less influential on birth outcomes and involve larger buffer zones. Nonetheless we found that measures of greater land cover related to larger newborn head circumference and that measures of land cover diversity associated with longer birth length. Future studies of greenspace should be cognizant of differences among metrics used to quantify residential greenspace exposure, and the potential need for broader spatial scales in studies focused on rural areas.

## Conflict of Interest

The authors declare no conflicts of interest relevant to this study.

## Supporting information

Supporting Information S1

## Data Availability

Health data for this research are considered protected health information and are not publicly available. Users wishing to negotiate access to the data under the terms of the Dartmouth CPHS and the Dartmouth Institutional Review Board should contact the principal investigator, Dr Margaret Karagas. Geospatial data (land cover, impervious surface area, and tree canopy cover) are available from Multi‐Resolution Land Characteristics Consortium (2021).
