# Supplementary material for: Greenspace and Land Cover Diversity During Pregnancy in a Rural Region, and Associations With Birth Outcomes
Source: Geohealth. 2024 Jan 23;8(1):e2023GH000905. doi: 10.1029/2023GH000905 (PMC10804422; doi:10.1029/2023GH000905)
Supplement: Supplementary file 1 — Supporting Information S1 [file GH2-8-e2023GH000905-s001.pdf]

GeoHealth

Supporting Information for

Greenspace and land cover diversity during pregnancy in a rural region, and associations with birth outcomes

Jonathan W. Chipman 1, Xun Shi 1, Diane Gilbert-Diamond 2,3, Camilo Khatchikian 2, Emily R. Baker 4, Mark Nieuwenhuijsen 5, and Margaret R. Karagas 2,3

1 Department of Geography, Dartmouth College, Hanover, NH USA

2 Department of Epidemiology, Geisel School of Medicine at Dartmouth, Lebanon, NH, USA

3 Children's Environmental Health and Disease Prevention Research Center at Dartmouth, Hanover, NH, USA

4 Department of Obstetrics and Gynecology, Dartmouth Hitchcock Medical Center, Lebanon, NH, USA

5 Centre for Research in Environmental Epidemiology, ISGlobal, Barcelona, Spain

Corresponding author: Jonathan W. Chipman (jonathan.w.chipman@dartmouth.edu)

Contents of this file

Figure S1
Table S1
Sensitivity Analysis: Alternative Methods and Data
Table S2
References

Introduction

Figure S1 shows the correlation between each pair of greenspace metrics, including all combinations of metric and buffer radius. Table S1 provides more details about the eight models that are highlighted as significant ($p < 0.05$) in Table 3.

	LC_0100	LC_0250	LC_0500	LC_1000	LC_2000	LC_3000	IS_0100	IS_0250	IS_0500	IS_1000	IS_2000	IS_3000	TC_0100	TC_0250	TC_0500	TC_1000	TC_2000	TC_3000	VI_0100	VI_0250	VI_0500	VI_1000	VI_2000	VI_3000	Div_0100	Div_0250	Div_0500	Div_1000	Div_2000	Div_3000	Dist_CL
LC_0100		88	74	62	52	46	-98	-84	-70	-60	-50	-44	47	54	51	45	34	29	63	59	53	47	38	33	-34	-40	-44	-39	-32	-27	9
LC_0250			91	79	68	60	-90	-99	-89	-77	-66	-58	43	55	56	53	44	39	60	68	64	58	49	43	-23	-34	-42	-41	-37	-33	9
LC_0500				93	82	74	-78	-92	-99	-92	-80	-73	36	47	56	57	51	46	53	66	71	67	60	54	-17	-25	-38	-43	-42	-39	8
LC_1000					92	84	-67	-82	-94	-99	-91	-83	31	38	50	59	57	52	47	58	66	72	67	60	-11	-16	-28	-42	-46	-43	6
LC_2000						95	-56	-71	-84	-93	-99	-95	25	31	43	54	62	60	40	51	59	67	72	68	-9	-11	-20	-35	-51	-51	5
LC_3000							-49	-63	-77	-85	-95	-99	22	27	38	48	59	63	35	46	54	62	70	73	-7	-8	-15	-30	-49	-55	3
IS_0100								89	75	65	54	48	-48	-54	-52	-46	-36	-31	-65	-62	-56	-50	-41	-36	32	37	42	38	32	28	-7
IS_0250									92	81	71	63	-42	-53	-55	-52	-45	-40	-60	-69	-66	-60	-51	-46	20	29	37	38	35	32	-7
IS_0500										94	84	77	-35	-45	-54	-55	-51	-46	-52	-65	-71	-68	-60	-55	14	21	32	38	38	37	-7
IS_1000											93	85	-30	-36	-48	-57	-56	-51	-45	-57	-66	-71	-67	-60	9	13	24	37	42	40	-5
IS_2000												96	-24	-30	-41	-52	-60	-58	-39	-50	-58	-66	-71	-68	7	9	16	31	46	47	-3
IS_3000													-21	-26	-37	-46	-58	-61	-34	-45	-53	-61	-69	-72	6	6	13	27	45	51	-2
TC_0100														-2	8	16	16	15	25	24	23	22	19	17	-15	-15	-15	-15	-16	-16	9
TC_0250															86	63	43	34	55	58	51	41	32	28	-28	-49	-58	-49	-35	-27	-5
TC_0500																85	62	50	49	58	61	55	45	40	-22	-44	-66	-63	-49	-40	-1
TC_1000																	82	68	43	52	59	65	59	52	-15	-32	-55	-73	-65	-55	0
TC_2000																		92	36	44	52	63	70	67	-7	-17	-34	-57	-76	-72	0
TC_3000																			31	39	47	57	69	73	-3	-11	-25	-46	-71	-79	1
VI_0100																				87	75	64	53	46	-26	-34	-40	-39	-36	-31	4
VI_0250																					92	78	65	56	-22	-34	-44	-46	-42	-37	4
VI_0500																						91	77	68	-18	-29	-45	-53	-50	-44	4
VI_1000																							90	80	-14	-21	-38	-57	-60	-55	3
VI_2000																								94	-10	-14	-27	-49	-68	-68	1
VI_3000																									-8	-11	-21	-41	-64	-73	3
Div_0100																										68	43	25	14	10	-1
Div_0250																											76	47	26	18	0
Div_0500																												75	46	33	-3
Div_1000																													78	61	-4
Div_2000																														91	-5
Div_3000																															-6
Dist_CL																															

Figure S1. Pearson's correlation coefficient *r* (x100) for all pairs of 31 greenspace metrics. Red grid cells indicate |r| ≥ 0.80.

Table S1. Regression details for models with greenspace p-values < 0.05 (highlighted in Table 3). SE = standard error of *β^*; RMSE=root mean squared error; MAE=mean absolute error. Slight differences in degrees of freedom for the t-statistic are due to the cross validation process, and to the presence of one additional variable (delivery type) in the models for head circumference.

Predictor	Outcome	*β^ (95% CI)*	SE	t statistic	p-val	RMSE	r^2	MAE
Div_1000	hght_z_birth	0.36 (0.07, 0.65)	0.15	t(1420)= 2.44	0.015	1.618	0.022	1.167
Div_2000	hght_z_birth	0.47 (0.15, 0.79)	0.16	t(1420)= 2.89	0.004	1.618	0.022	1.165
Div_3000	hght_z_birth	0.5 (0.17, 0.84)	0.17	t(1420)= 2.97	0.003	1.618	0.022	1.166
LC_2000	headcirc_ z_birth	1.08 (0.01, 2.15)	0.55	t(1418)= 1.97	0.049	2.924	0.015	1.374
LC_3000	headcirc_ z_birth	1.32 (0.09, 2.56)	0.63	t(1418)= 2.1	0.036	2.924	0.015	1.374
IS_3000	headcirc_ z_birth	-2.24 (-4.38, -0.1)	1.09	t(1418)= -2.05	0.04	2.924	0.015	1.374
TC_0100	headcirc_ z_birth	0.58 (0.01, 1.14)	0.29	t(1418)= 2.01	0.045	2.925	0.015	1.372
TC_0250	headcirc_ z_birth	-0.5 (-0.93, -0.07)	0.22	t(1419)= -2.27	0.023	2.926	0.015	1.371

Sensitivity Analysis: Alternative Methods and Data

As described in Section 2 of the main text of this paper (Materials and Methods), the birth cohort dataset had missing values for some covariates in some data records (Table 1). These were imputed using the Multivariate Imputation by Chained Equations algorithm (MICE), which is based on predictive mean matching for continuous data, and logistic regression, polytomous logistic regression, and proportional odds for binary and categorical data (Van Buuren and Groothuis-Oudshoorn, 2011). In addition, there were substantial numbers of outliers in some variables, which suggested the use of a regression algorithm that is particularly robust to outliers: the Robust Fitting of Linear Models (rlm) algorithm (Hampel et al., 2011; Huber, 2004; Marazzi, 1993).

To ensure that the results reported in the main text of this paper are not an artifact of the particular methods used, we performed a sensitivity analysis using (a) only the 827 birth cohort data records with no missing values in any of the covariates, to eliminate the need for imputation; and (b) the more widely-used glm function in R for generalized linear models (Dobson, 1990). The results were broadly similar to those of the original analysis, although only two models achieved significance at $\alpha = 0.05$, possibly due to the decreased sample size (n = 827 vs 1440 in the original study). The model for length z-score with land cover diversity in a 3000 m radius had a p-value of 0.0475; this model

had the highest significance in the original analysis (p = 0.0030). The length z-score model with land cover diversity in a 500 m radius also was significant in this sensitivity analysis (p = 0.0388), despite not quite achieving significance at α = 0.05 in the original work (p = 0.0556). No other models were significant in this alternative analysis, confirming the original finding of a general scarcity of significant relationships outside of the land cover diversity & length z-score models.

We also conducted an assessment of two additional birth outcomes, to determine whether either preterm births or low birth weights were significantly associated with any of the greenspace metrics. Both of these were modeled using binomial versions of the generalized linear model algorithm in R, for the full set of 1440 birth cohort participants, and using each of the 31 greenspace metrics. As shown in Table S2, none of these 62 models were significant at α = 0.05. Note, for comparison, that two related birth outcomes discussed in the main text of this paper (gestational age and weight z-score) also were not significantly associated with any of the greenspace metrics (Table 3).

Table S2. Model significance (p-value) for 31 greenspace metrics and two birth outcomes: preterm birth and low birth weight.

Greenspace metric	Preterm birth (y/n) p-value	Low birth weight (y/n) p-value
LC_0100	0.106	0.251
LC_0250	0.385	0.385
LC_0500	0.852	0.705
LC_1000	0.763	0.951
LC_2000	0.762	0.89
LC_3000	0.94	0.579
IS_0100	0.251	0.449
IS_0250	0.534	0.517
IS_0500	0.916	0.682
IS_1000	0.815	0.876
IS_2000	0.779	0.846
IS_3000	0.907	0.546
TC_0100	0.509	0.475
TC_0250	0.676	0.19
TC_0500	0.684	0.214
TC_1000	0.595	0.521
TC_2000	0.975	0.986
TC_3000	0.705	0.678
VI_0100	0.555	0.215
VI_0250	0.695	0.23
VI_0500	0.842	0.423

VI_1000	0.785	0.743
VI_2000	0.791	0.704
VI_3000	0.774	0.777
Dist_CL	0.861	0.282
Div_0100	0.791	0.629
Div_0250	0.712	0.316
Div_0500	0.327	0.233
Div_1000	0.885	0.933
Div_2000	0.325	0.903
Div_3000	0.301	0.732

References

Dobson, A. J., 1990. *An Introduction to Generalized Linear Models*. Chapman and Hall, London.

Hampel, F.R., Ronchetti, E.M., Rousseeuw, P.J., Stahel, W.A., 2011. *Robust Statistics: The Approach Based on Influence Functions*. John Wiley & Sons.

Huber, P.J., 2004. *Robust Statistics*. John Wiley & Sons.

Marazzi, A., 1993. Algorithms, Routines, and S-Functions for Robust Statistics. CRC Press.
Markevych, I., Schoierer, J., Hartig, T., Chudnovsky, A., Hystad, P., Dzhambov, A.M., de Vries, S., Triguero-Mas, M., Brauer, M., Nieuwenhuijsen, M.J., Lupp, G., Richardson, E.A., Astell-Burt, T., Dimitrova, D., Feng, X., Sadeh, M., Standl, M., Heinrich, J., Fuertes, E., 2017. Exploring pathways linking greenspace to health: Theoretical and methodological guidance. Environ. Res. 158, 301–317. https://doi.org/10.1016/j.envres.2017.06.028

Van Buuren, S., Groothuis-Oudshoorn, K., 2011. mice: Multivariate Imputation by Chained Equations in R. *J. Stat. Softw.* 45, 1–67. https://doi.org/10.18637/jss.v045.i03
